# Detection of Lymph Node Metastases by Ultra-pH-Sensitive Polymeric Nanoparticles

**DOI:** 10.7150/thno.41239

**Published:** 2020-02-10

**Authors:** Zachary T. Bennett, Qiang Feng, Justin A. Bishop, Gang Huang, Baran D. Sumer, Jinming Gao

**Affiliations:** 1 Department of Pharmacology, Harold C. Simmons Comprehensive Cancer Center, University of Texas Southwestern Medical Center, Dallas, Texas, USA.; 2 Department of Pathology, University of Texas Southwestern Medical Center, Dallas, Texas, USA; 3 Department of Otolaryngology, University of Texas Southwestern Medical Center, Dallas, Texas, USA

**Keywords:** Lymph node metastasis, Image-guided surgery, Polymeric micelle

## Abstract

Lymph node (LN) dissection followed by histological analysis is the current standard for diagnosis of LN metastasis but the method suffers from patient morbidity and low sensitivity of detection. Ultra-pH sensitive (UPS) nanoparticles show remarkable accuracy in the delineation of primary tumor margins for precision cancer surgery. Herein we investigate the effectiveness of UPS nanoparticles to detect cancer-involved LNs.

**Methods**: We synthesized a series of indocyanine green (ICG) conjugated UPS nanoparticles with distinct pK_a_ (UPS_5.3_, UPS_6.1_, and UPS_6.9_). Systemically administered UPS-ICG nanoparticles in the 4T1.2-BALB/cj mouse model were imaged with real-time, near-infrared fluorescence (NIRF) to guide removal of LNs. *Ex vivo* imaging of gross tissue enabled quantification of fluorescence intensity. Histological analysis was used as the gold standard diagnostic test.

**Results**: Macrophage uptake of UPS nanoparticles elevates the background signal in benign LNs. However, cancer foci within LNs show distinctive clustering of UPS-ICG fluorescence. UPS_5.3_ achieves accurate detection of metastatic LNs as shown by a receiver operating characteristic (ROC) area under the curve (AUC) of 0.96 ± 0.03. UPS_6.1_ and UPS_6.9_ offer decreased discriminatory power at ROC AUC of 0.73 ± 0.1 and 0.88 ± 0.07, respectively.

**Conclusions**: All UPS compositions show cancer-specific discrimination of metastatic LNs over benign LNs with the best outcomes from UPS_5.3_. Detection of micro-metastatic LNs (cancer foci < 2 mm) remains a challenge. This study provides information on the detection of LN status for image-guided resection of metastatic LNs.

## Introduction

In many solid cancers, lymph node (LN) status is the single most important prognostic indicator of patient survival 1-5. Regional metastasis to axillary LNs in breast cancer decreases 5-year survival by 28-40% 6. Determination of LN status is critical to stage cancer accurately and to direct therapy. Often LN metastases are detectable with palpation and intraoperative visualization or by noninvasive imaging, but low-volume metastatic disease is imperceptible with these methods 7. Therefore, the gold standard to diagnose LN status is a regional LN dissection. The goals of a regional LN dissection are therapeutic removal of the cancer-involved LN(s) and diagnostic sampling of enough relevant LNs to improve staging 8. This is an effective technique as 16% of specimens from axillary LN dissection have occult disease detected on final pathology only 9. However, patients often suffer from lymphedema, pain, numbness and a 2.9-fold increased risk of impaired arm use with regional LN dissection 10, 11. To limit the significant morbidity, the sentinel lymph node biopsy (SLNB) is an alternative technique.

In SLNB, intratumoral administration of contrast medium (e.g., methylene blue) identifies the most proximal LNs to the primary tumor 12. Removal and histopathologic analysis of these sentinel LNs increases the chance of detecting LN metastases compared to noninvasive imaging alone and causes less morbidity than regional LN dissections. However, SLNB still has a false negative rate of 12.6-51.6% 13, 14. Since only the sentinel LNs are removed, skip metastases and alterations in lymphatic drainage can result in false negative diagnoses 15. In addition, disseminated cells may colonize distant LNs outside the regional basin 16, 17. Therefore, an intraoperative method for visualizing LNs using systemically administered contrast medium could reduce the false negative rate of SLNB. This could allow removal of at-risk LNs, which may harbor micro-metastases, without the significant morbidity of a full lymphadenectomy.

While there are few functional indicators of LN metastasis, deregulated cellular metabolism is a universal hallmark of cancer 18. Enhanced aerobic glycolytic activity, known as the “Warburg Effect”, is observed in many cancers and exploits ATP production from the oxidation of glucose to lactate 19. Downstream metabolites, such as lactic acid, are secreted extracellularly and lead to increased acidosis in the tumor microenvironment 20. Additionally, recent studies reported the metabolic profile of metastatic LNs has elevated levels of CO_2_ compared to benign LNs due to upregulated consumption of fatty acids and an enhanced oxygen consumption rate by metastasized cancer cells 21. A potential effect of the elevated CO_2_ and tissue hypoxia includes further contribution to cancer acidosis within the metastatic LNs, serving as a potential biomarker for detection 22.

**Figure 1:**
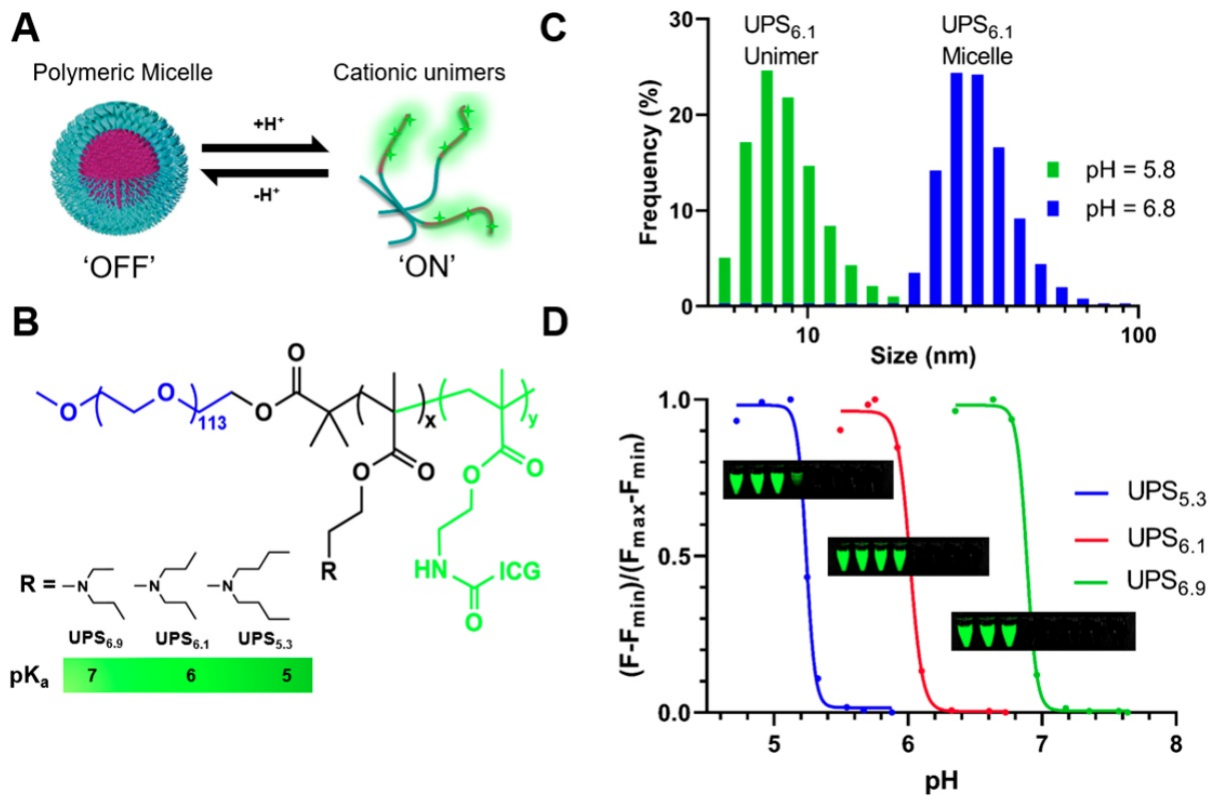
** Binary fluorescence responses of the ultra-pH-sensitive (UPS) polymeric micelle probes towards discrete pH-thresholds.** (A) UPS micelles are self-assembled nanoparticles that disassemble into unimers in response to threshold proton concentrations. (B) Structures of amphiphilic block copolymers enable cooperative pH response at specific pK_a_. (C) Dynamic light scattering shows distinct populations of sizes for unimers (pH below pK_a_) and micelles (pH above pK_a_) for a representative UPS_6.1_ nanoparticle. (D) Non-linear amplification of fluorescence intensity shows ultra-pH-sensitive response to environmental pH signals. Inset tubes show the near-infrared visualization of UPS_5.3_-ICG (top), UPS_6.1_-ICG (middle), and UPS_6.9_-ICG (bottom) as a function of pH. Each tube corresponds with a dot on the graph.

Our laboratory has developed a class of ultra-pH-sensitive (UPS) micellar nanoparticles which amplify near-infrared fluorescent (NIRF) emissions in response to subtle changes in pH (Figure 1A) 23. Ultra-sensitivity describes an output response that is more sensitive to an input stimulus than a Michaelis-Menten response 24, 25. In the UPS nanoparticle system, intramolecular cooperative forces drive self-assembly with a large Hill coefficient (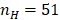) 26. We have shown the 'transistor-like' binary ON/OFF phenomenon in the UPS system to delineate tumor margins at high precision for image-guided resection. Even visibly occult, murine mammary carcinomas (1-5 mm^3^) are detectable under NIRF imaging 27. Moreover, an *irreversible* 'capture and integration' mechanism converts dynamic, analog tumor acidotic signals into a binary, stable reporter output for improved tumor detection 28. We theorize this time-integrated signal amplification coupled with the tumor specific detection is scalable towards low-volume disease such as occult LN metastases. In this study, we investigate the utility of UPS polymeric micelles towards detection of metastatic LNs in a murine breast cancer model.

## Results

### UPS nanoparticles show cooperative fluorescence response to environmental pH

We synthesized three UPS block copolymers with discrete pH-transitions to cover a range of pH response (UPS_5.3_, UPS_6.1_, and UPS_6.9_; each subscript indicates the apparent pK_a_ value) (Figure 1B, Table S1-3). In particular, the amphiphilic block copolymer UPS_6.1_ has a pK_a_ at 6.1. At pH-values above the pK_a_, UPS_6.1_ self-assembles into 24.0 ± 2.1 nm micelles (Figure 1C, Table S3). Below pH-values of 6.1, protonation of polymer chains causes micelle disassembly into 4.9 ± 1.2 nm unimers (Figure 1C). UPS_5.3_ (28.5 ± 1.5 nm) and UPS_6.9_ (23.4 ± 2.5 nm) also have sharp pH-dependent micelle-to-unimer transitions as well (Table S3, Figure S1C). The comparable nanoparticle size (23-28 nm) and identical PEG length (5 kDa) between micelle compositions are important to keep size and surface chemistry consistent in LN targeting 29, enabling the specific evaluation of pH-thresholds in the detection of LN metastases.

To report local pH values, we conjugated each polymer with indocyanine green (ICG), a fluorophore that is approved by the FDA and compatible with clinical, NIRF imaging systems. Each UPS-ICG nanoparticle shows comparable copies of dye per polymer (Table S3, Figure S1A). However, in the micelle state at pH 7.4, homoFRET-induced quenching abolishes the ICG fluorescence signal. At pH below the pK_a_, UPS micelles disassemble into individual unimers and amplify fluorescence intensity over 50-fold within a 0.3 pH span (Figure 1D, Table S2). UPS displays binary encoding of pH-thresholds by NIRF (Figure 1D, S1A-B, Table S2). This 'digital' signal represents fluorescence activation as a discrete value (ON = 1, OFF = 0) at different pH-threshold.

### Real-time systemic lymphatic mapping in tumor naïve mice guides resection of LNs

**Figure 2:**
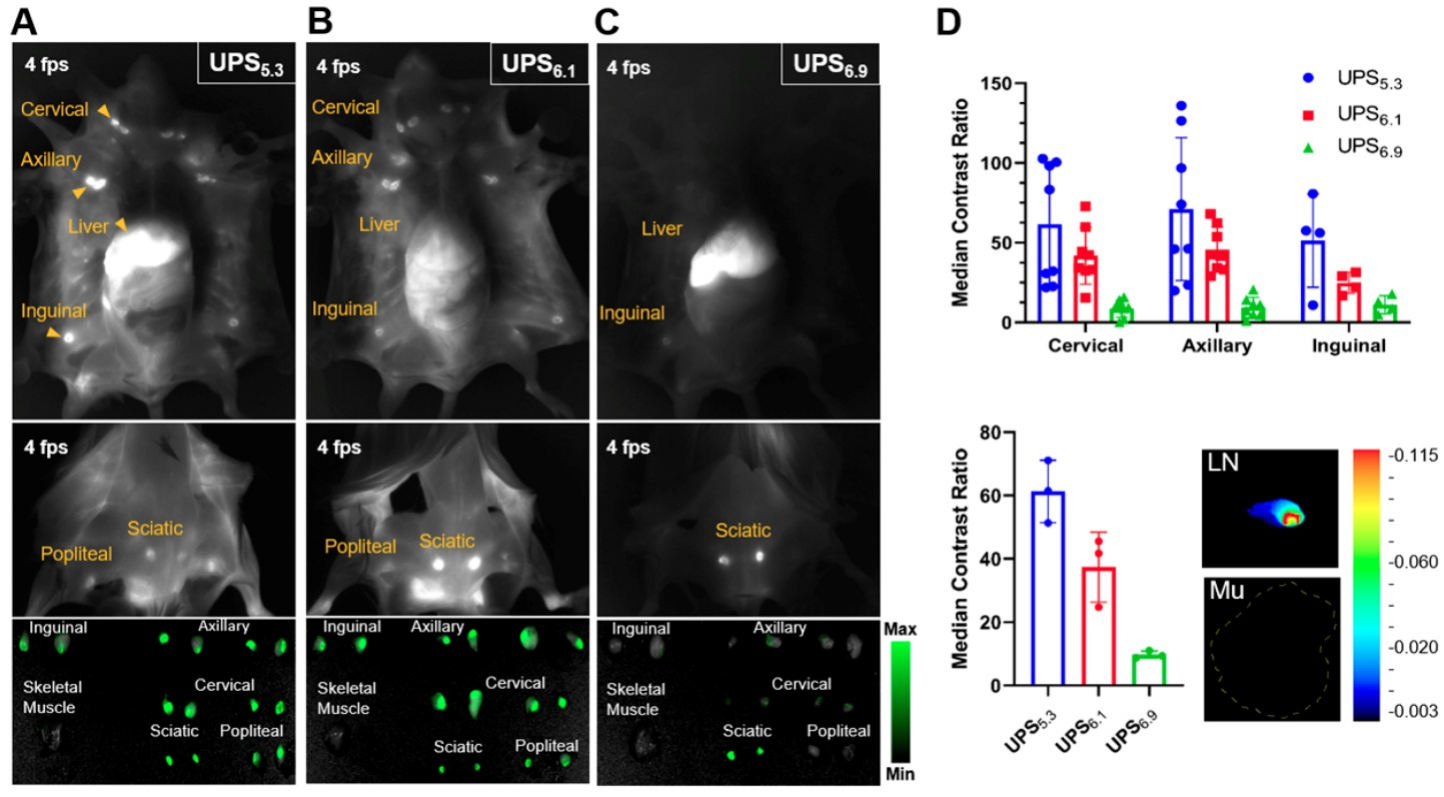
** Whole body near-infrared fluorescence imaging of dissected, tumor-naïve BALB/cj mice enables image-guided resection of LNs in real-time.** (A) UPS_5.3_-ICG and (B) UPS_6.1_-ICG delineate all the superficial LNs, enabling imaged guided resection. (C) UPS_6.9_-ICG fluorescence is mostly sequestered to the liver. Image-guided resection of LNs is not permissible. (D) Median fluorescence intensity of LNs is normalized to that of skeletal muscle (Mu). The median CR of anatomical LN group shows dependence on the pK_a_ of polymeric micelle. UPS_5.3_ shows the highest intensity within each anatomical group of LNs.

We intravenously administered each polymeric nanoparticle formulation in tumor-naïve BALB/cj mice to evaluate whole-body lymphatic mapping. NIRF imaging visualizes dissected mice, clearly delineating LNs in the UPS_5.3_ and UPS_6.1_ administered animals (Figure 2A-B). This delineation facilitates image-guided resection of all superficial LNs in real-time. Quantitative imaging of resected tissue *ex vivo* with the LICOR Pearl shows comparable ICG signals from different anatomical groups of LNs. We calculated the median contrast ratio (CR) for all LN tissues:


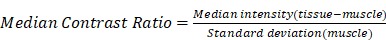


LN fluorescence is amplified with a pan-LN median CR of 63.3 for UPS_5.3_ and 39.9 for UPS_6.1_ (Figure 2D). However, the UPS_6.9_ median CR value is 10.7 (Figure 2D), which is significantly lower.

To explain the differences between micelle compositions in LN targeting, we performed a pharmacokinetics study, evaluating fluorescence in tumor-naïve BALB/cj blood plasma after intravenous injection (Figure S2A). UPS_6.9_ is quickly cleared from the blood compared to UPS_5.3_ and UPS_6.1_ (Figure S2A). In addition, UPS_6.9_-ICG has low ON/OFF ratio after acidification of blood plasma, indicating UPS_6.9_ disassembles 24 h after intravenous injection (Figure S2B). All nanoparticles, including UPS_6.9_, are stable over 24 h with high ON/OFF ratios during incubation in normal mouse serum (Figure S3). We attribute the low ON/OFF ratio of UPS_6.9_ to the fast clearance of the nanoprobes in the liver (Figure S2C), which results in lower serum concentration and increased thermodynamic propensity to disassemble.

### LN-resident macrophages internalize UPS polymeric micelles

**Figure 3:**
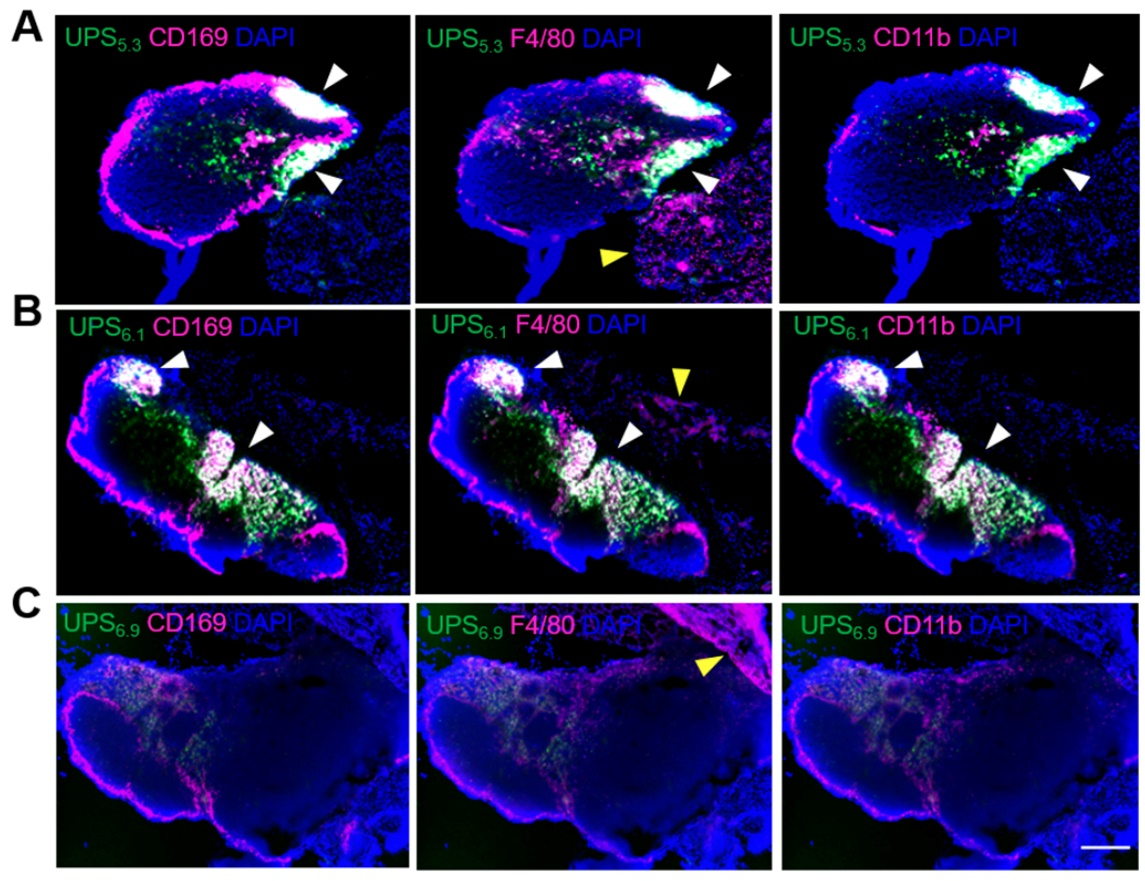
** Co-localization of UPS nanoparticles with macrophage sub-populations shows uptake of micelles by lymph node resident macrophages.** (A) UPS_5.3_-ICG co-localizes with CD169 (left), F4/80 (middle), and CD11b (right), but the co-localization is limited within the lymph node. White arrows show co-localization between positive cells and ICG fluorescence. Yellow arrows show staining of F4/80 cells without presence of ICG fluorescence. (B) The pattern of UPS_6.1_-ICG co-localization with macrophage mirrors that of UPS_5.3_-ICG. (C) UPS_6.9_-ICG fluorescence intensity is much lower than UPS_5.3_-ICG and UPS_6.1_-ICG. All panels show phagocytosis of nanoparticles by the macrophages in the lymph node but not those in the surrounding tissue. Scale bar is 200 μm.

While NIRF imaging delineates all superficial LNs, the lymphotropic delivery mechanism is unclear. Because phagocyte-containing reticuloendothelial systems (e.g., liver, spleen) have increased fluorescence intensity, we theorized LN-resident macrophages are responsible for the uptake of UPS micelles, leading to amplification of ICG fluorescence signals. To investigate this hypothesis, we utilized multiplexed immunohistochemistry (IHC) staining of distinct macrophage populations along with visualization of UPS nanoparticle uptake 30, 31. UPS_5.3_-ICG and UPS_6.1_-ICG fluorescence signals appear in distinct regions in the LN (Figure 3A-B). These regions show significant overlap with LN-resident macrophages. Specifically, CD169^+^/F4/80^+^/CD11b^+^ macrophages co-localize with UPS_5.3_-ICG fluorescence (Figure S4). These cells share the same biomarkers as LN-resident macrophage 26. Additionally, ICG fluorescence does not overlap with F4/80^+^ macrophages in the adjacent tissues surrounding the LN, supporting the assumption of LN-specific delivery (Figure 3A-B), indicating only LN-resident macrophage sequester UPS nanoparticles. Moreover, microscopic analysis shows UPS_6.9_ has low accumulation in LNs from tumor-naïve mice (Figure 3C, same imaging condition as 3A-B), which is in accordance with macroscopic imaging data (Figure 2C). The lower signal in benign LNs by UPS_6.9_ could potentially offer low background for metastatic LN detection.

### Detection of metastatic LNs in tumor-bearing mice

**Figure 4:**
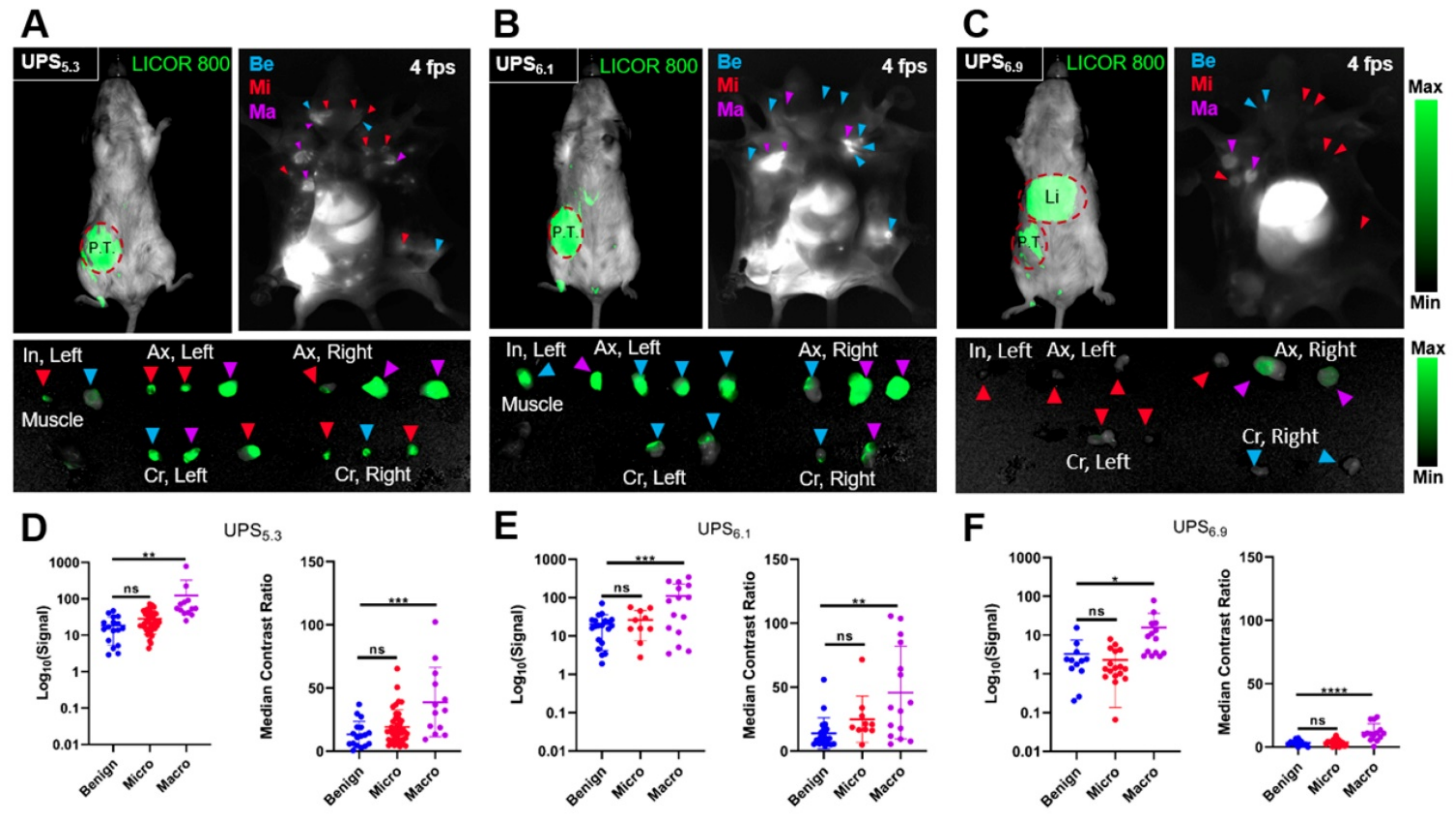
** Detection of metastatic lymph nodes with verification by histological examination.** (A) A representative 4T1.2-bearing BALB/cj mouse administered with UPS_5.3_-ICG shows NIRF detection of the primary tumor (P.T.) with whole body imaging as well as delineation of benign (Be), micro-metastatic (Mi), and macro-metastatic (Ma) LNs, enabling image-guided resection of inguinal (In), axillary (Ax), and cervical (Cr) LNs. (B) NIRF imaging of UPS_6.1_-ICG administered mice shows delineation of the primary tumor and LNs, with the benign LNs appearing nearly as bright as the metastatic LNs. (C) UPS_6.9_-ICG accumulates at much higher intensity within the liver (Li). Some macro-metastatic LNs are delineated, but many micro-metastatic LNs are undetectable. (D) UPS_5.3_ signal and median CR of classified tissue shows significance between metastatic and benign LNs. Statistical analysis is done with one-way ANOVA followed by Tukey's multiple comparisons test (*P < 0.033, **P < 0.0021, ***P < 0.0002, ****P < 0.0001). (E) UPS_6.1_ signal and median CR of classified tissue shows significance between macro-metastatic and benign LNs, but the variance in the macro-metastatic distribution is high. (F) UPS_6.9_ signal and median CR of classified tissue shows significance between macro-metastatic and benign LNs. The signal variable is much lower in intensity compared to UPS_5.3_ and UPS_6.1_.

**Figure 5:**
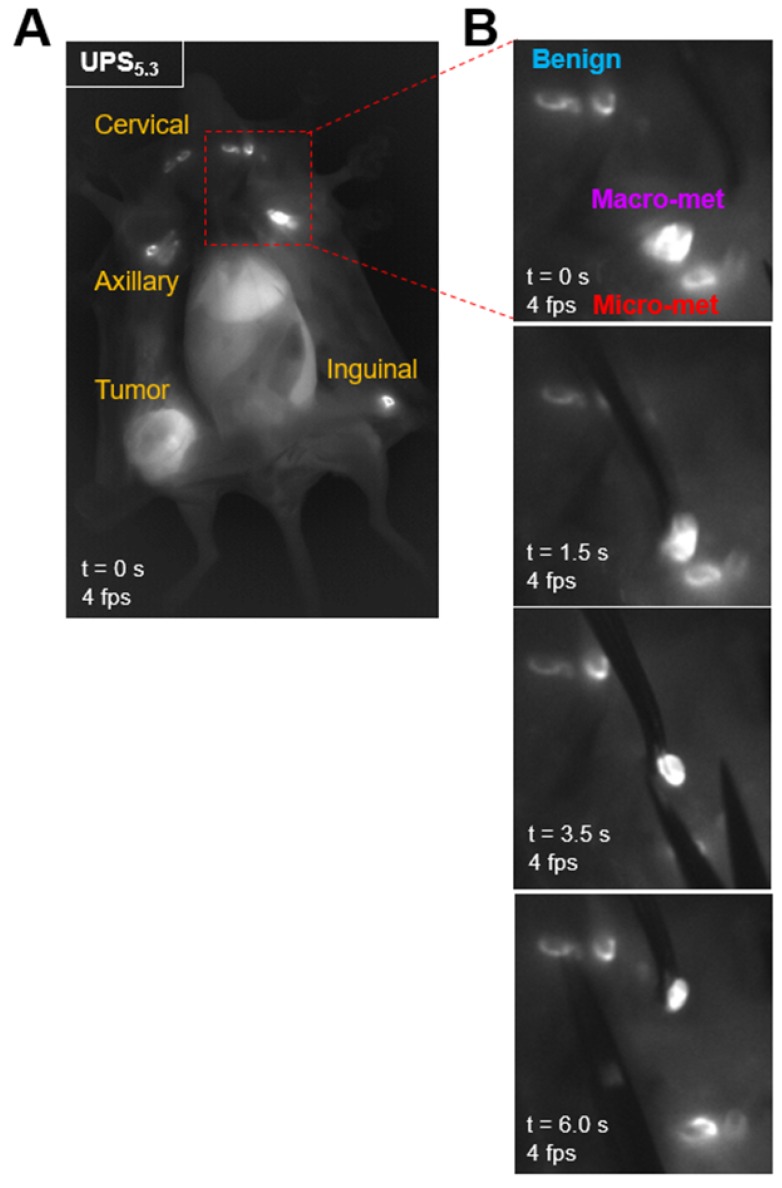
** Resection of metastatic lymph nodes in real-time using NIR fluorescence guidance.** (A) A 4T1.2-bearing BALB/cj mouse is intravenously injected with UPS_5.3_-ICG, euthanized, dissected and imaged with the near-infrared camera at 4 fps. All superficial LNs and the primary tumor are delineated. (B) LNs in anatomical regions are visible. A macro-metastatic LN shows increased fluorescence intensity, distinct spatial accumulation of fluorescence, and is larger than other LNs. This LN is resected using the guidance of the NIR fluorescence as feedback. Sampling of other at-risk LNs in the same regional basin is possible. All LN pathology is confirmed by histological examination.

We quantified the differences in fluorescence intensity of metastatic LNs against benign LNs using the syngeneic 4T1.2-BALB/cj murine model. UPS_5.3_, UPS_6.1_, or UPS_6.9_ nanoparticles were intravenously administered at the same dose (1.0 mg/kg) for systemic detection of LN metastases. NIRF imaging of live mice by the LICOR Pearl, after 24 h circulation, showed fluorescence emission within the primary tumor but not metastatic LNs (top left panels, Figure 4A-C). In contrast, NIRF imaging of dissected mice shows accumulation in LNs in addition to primary tumors (top right panels, Figure 4A-C). UPS_5.3_ and UPS_6.1_ administered animals show bright fluorescence signal in all superficial LNs (Figure 4A-B). UPS_6.9_ administered animals show micelle accumulation in enlarged LNs (Figure 4C). Real-time fluorescence imaging enabled guided resection of all LNs (Figure 5A-B). Macro-metastatic LNs are often distinct in fluorescence intensity, spatial pattern, and size from other LNs, enabling precision resection of these LNs (Figure 5B).

We quantified the median contrast ratio for all resected tissue (Equation 1). We also used the LICOR Signal to quantify the total fluorescence intensity from a region of interest (ROI). Each variable conveys distinct information. Median CR evaluates the pixel-based, median fluorescence intensity of LNs whereas LICOR signal reports the summated fluorescence intensity of the LN tissue. We evaluated both variables in statistical analysis of grouped tissue. Histological examination of LNs allowed for grouping of tissue based on pathology. We classified LNs as either benign, micro-metastatic (cancer foci < 2 mm), or macro-metastatic (cancer foci > 2 mm). We grouped median CR and LICOR signal values accordingly (Figure 4D-F). There is a significant difference between benign and macro-metastatic groups (Figure 4D-F). However, no micelle groups displays a significant difference between benign and micro-metastases.

### UPS nanoparticles accumulate within the cancer foci of metastatic LNs

**Figure 6:**
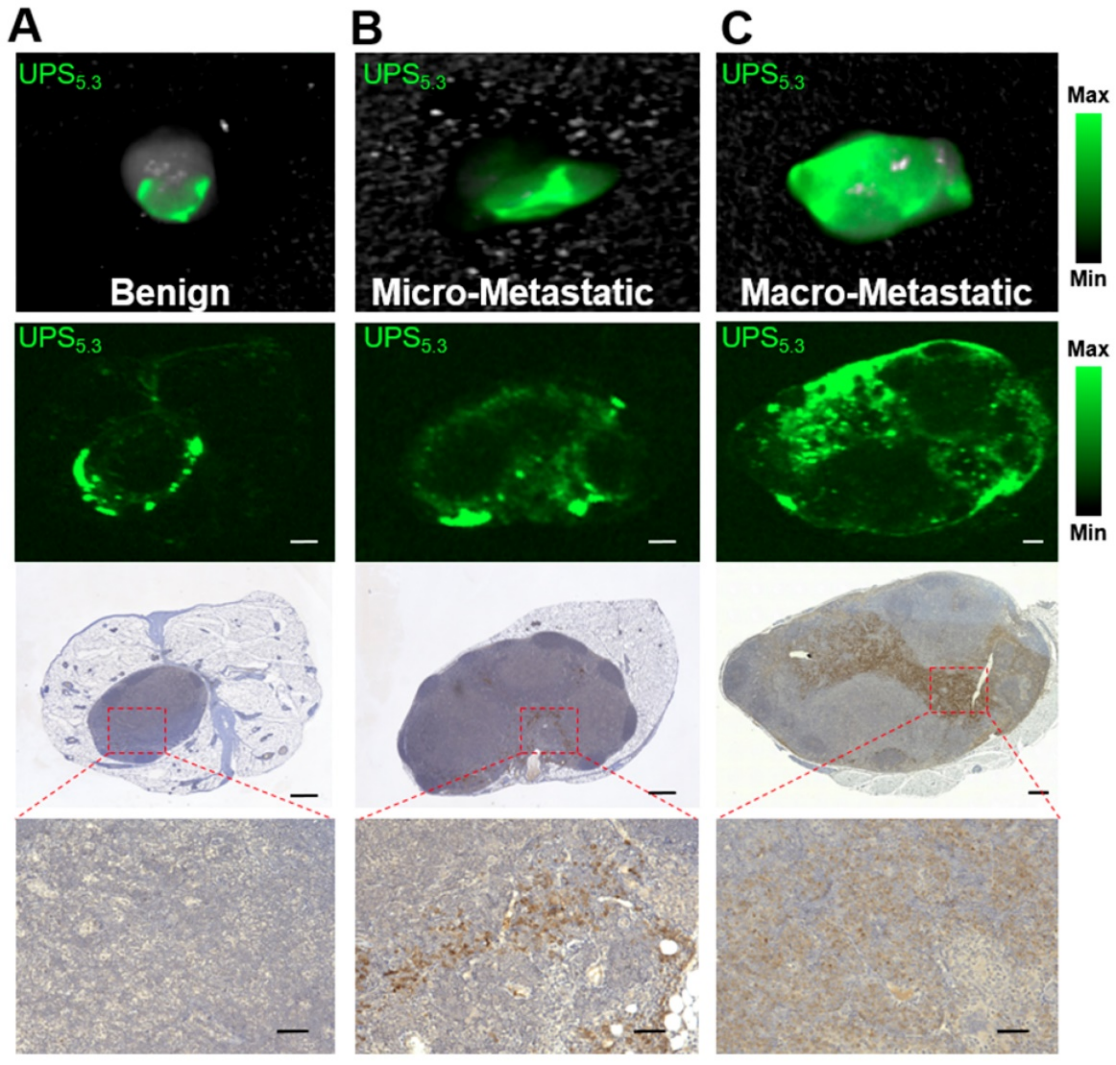
** Discrimination of metastatic from benign lymph nodes based on ICG patterns.** (A) NIRF imaging of benign LNs show ICG fluorescence at the periphery of the nodes. H&E histology and negative pan-cytokeratin stain were used to verify the lack of cancer foci. (B) Micro-metastatic LNs show some UPS_5.3_-ICG fluorescence in the core of the LN. (C) Macro-metastatic LNs show a broad pattern of ICG fluorescence across the enlarged LN tissue. Pattern of ICG fluorescence correlates with dense cytokeratin staining. Upper and lower scale bars are 300 and 50 μm, respectively.

In addition to differences in fluorescence intensity, we identified different patterns of fluorescence signal between benign LNs and macro-metastatic LNs. Benign LNs display a 'halo' of UPS_5.3_-ICG intensity by both real-time imaging and *ex vivo* imaging (Figure 5A-B, 6A). Histological analysis confirms no pan-cytokeratin clusters in this LN subset (Figure 6A). Moreover, microscopic imaging confirms the accumulation of UPS nanoparticles at the edges of LN tissue (Figure 6A). This pattern is also apparent with UPS_6.1_ and UPS_6.9_ administered animals (Figure S5). The peripheral distribution of UPS_5.3_ nanoparticles in benign LNs colocalizes with LN-resident macrophages in the LN sinusoids (Figure S6A). These results are in agreement with the fluorescence localization in tumor-naïve LNs (Figure 3). However, in benign LNs from tumor-bearing mice, CD11b^+^ macrophages appear more motile within the surrounding tissue compared to the same population in tumor-naïve mice (Figure S6C).

Micro-metastatic LNs show a spectrum of fluorescence signatures. Fluorescence may localize to LN edges or show uniform fluorescence across small cancer foci. A mixed pattern with both fluorescence localization at edges and within pan-cytokeratin clusters is the most typical signature (Figure 6B). In contrast, macro-metastatic LNs display a broad pattern of fluorescence intensity (Figure 6C). Microscopic analysis shows the ICG signal overlaps mostly with anti-cytokeratin staining (Figure 6C), indicating cancer-specific accumulation of UPS unimers. We observed a similar result with the UPS_6.1_ administered group (Figure S7). Moreover, fluorescence intensity of metastatic LN tissue from the UPS_6.9_ group is decreased compared to UPS_6.1_ and UPS_5.3_ (Figure S7).

### ROC discrimination of metastatic LNs from benign LNs

**Figure 7:**
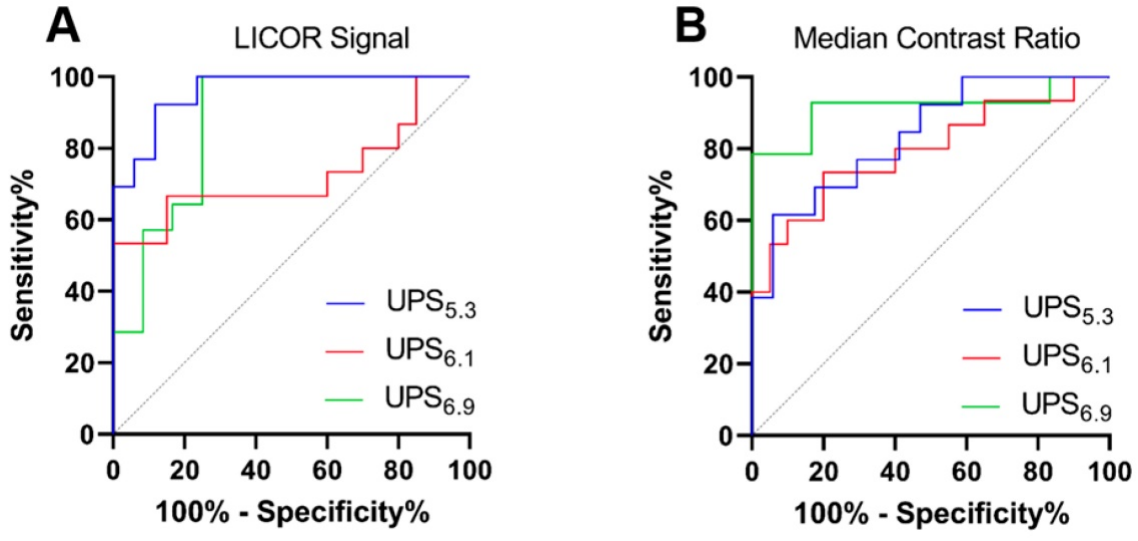
** Receiver operating characteristic (ROC) analysis of metastatic lymph node detection by UPS nanoparticles.** (A) ROC curves showing sensitivity and specificity of macro-metastatic LN detection using the LICOR signal of the whole node. UPS_5.3_ has an AUC of 0.96, indicating high discriminatory capabilities. (B) ROC analysis based on the median CR variable. UPS_6.9_ has higher discriminatory capability, but it has lower ICG signal as shown in Figure 4C.

We quantified the receiver operating characteristic (ROC) of macro-metastatic LN detection (Table 1). Quantifying tissue with size-dependent LICOR signal reveals UPS_5.3_ has high discriminatory power (AUC = 0.96; sensitivity = 92.3% and specificity = 88.2%) of macro-metastatic LNs over benign LNs (Figure 7A). Discrimination of benign LNs from macro-metastatic LNs is also feasible using median CR for each polymer (Figure 7B). UPS_6.9_ shows significant discrimination (AUC = 0.92; sensitivity = 78.6% and specificity = 100%) with median CR. Complete ROC analysis is presented in Table S1. Moreover, we quantified ROC of micro-metastatic LN discrimination (Table S2). The data indicates a lack of discrimination of micro-metastases over benign LNs with either median CR or LICOR signal (Figure S8).

## Discussion

Lymph nodes (LNs) are often the first site to which solid cancers metastasize. LN status is a part of the formal staging for all solid cancers because metastasis to LNs worsens prognosis. The gold standard for determination of LN status is removal and microscopic examination of all at-risk LNs. These LNs often drain lymph from the primary tumor. Their removal happens in bundles since precise identification of the small LNs is often difficult and variable within a given level 32. However, removal of multiple levels via a lymphadenectomy can lead to significant morbidity 11. Recent advances, such as the SLNB, enable determination of some at-risk LNs due to better understanding of lymphatic drainage anatomy 33. Yet, SLNB cannot identify many at-risk LNs, leading to false negative diagnoses 34. Therefore, more information on the location of both benign and cancer-involved LNs could further improve surgical targeting of the most relevant LNs for removal.

This study demonstrates the fluorescent detection of cancer foci within LNs by ultra-pH-sensitive (UPS) micelles. Three micelles (UPS_5.3_, UPS_6.1_, and UPS_6.9_) display accumulation in pan-cytokeratin positive cancer foci, resulting in detectable fluorescence signals (Figure 6C, S7). Quantification of fluorescence intensity reveals LICOR signal is an appropriate metric to achieve discrimination of LN metastasis, especially in the UPS_5.3_ group (Figure S8). Although LN-resident macrophage uptake of UPS nanoparticles causes background fluorescence, the resulting fluorescence intensity is quantifiably distinct from metastatic LNs. There appears to be a lower limit to the volume of disease that can be discriminated by fluorescence intensity; micro-metastatic LNs are not distinguishable from benign LNs. In our study, this size classification had a threshold at 2 mm.

In addition to fluorescence intensity alone, pattern-based discrimination of LN pathology is a potential second line of distinction. Benign LNs display a 'halo' of fluorescence most likely due to uptake of micelles by LN-resident macrophages (Figure 6A). Within these LNs, medullary sinus macrophage uptake would support the hypothesis of vasculature delivery of micelles to the LN 30. It appears these macrophages internalize micelles upon delivery to LNs and amplify the fluorescence within their acidic organelles 35. Conversely, metastatic LNs show a broad pattern of fluorescence throughout the LN cortex correspondent with cancer-foci (Figure 5A-B, 6C). This pattern of activation could be detectable by the surgeon during resection. There is potential to utilize both intensity and spatial localization of fluorescence to achieve greater discrimination of metastatic LNs.

Systemically administered polymeric nanoparticles enable real-time lymphatic mapping (Figure 2). Regional lymphatic mapping is a difficult clinical procedure due to LN multiplicity and unpredictable location 36. Often, locally administered contrast medium cannot detect all LNs in the regional basin. In addition to the technical challenges of precision LN resection, there is still clinical debate regarding the role of regional LN dissections following a positive sentinel LN biopsy in breast cancer 37. Our results indicate the delineation of all LNs by systemically administered nanoparticles. This could allow precise detection of at-risk LNs that may or may not have cancer but likely would benefit from surgical sampling (Figure 5B), potentially circumventing the need to dissect out an entire LN level for effective removal.

Biodistribution of micelles to LNs appears to be a critical parameter for discrimination of metastatic LNs. UPS_6.9_ has a lower blood half-life than UPS_6.1_ and UPS_5.3_ as shown by increased accumulation in the liver in both tumor-bearing and tumor-naïve mice (Figure 4, S2). Compared to UPS_6.1_ and UPS_5.3_, UPS_6.9_ shows decreased background fluorescence in LNs, enabling discrimination with improved median CR response (Figure 7B). To investigate further the effect of biodistribution and circulation time on LN metastasis detection, we included circulation times of 6 h and 72 h after intravenous administration of UPS_5.3_ nanoparticles. We found sinusoidal macrophage take up nanoparticles quickly as the 'halo' phenomenon is present in LNs from the 6 h group (Figure S9A). Moreover, it does not appear longer circulation time permits increased discrimination of LN metastasis (Figure S9C). Overall, the increased half-life of UPS_5.3_ enables comparatively better 'capture and integration' of ICG fluorescence within the lymph node metastasis microenvironment.

We successfully detected several locoregional metastases in soft tissues outside of LNs during our study (Figure S10). It is unclear if these are in-transit metastases from lymphatics or blood-borne distant metastases. However, metastases outside of the traditional LN basins and primary tumor margins are often difficult to identify and can cause local failure even with clear margins and LN dissection 38. Detection of locoregional or distant metastases may be improved with the use of systemically administered UPS micelles.

In conclusion, our study demonstrates functional imaging of acidosis in cancer-involved LNs using UPS nanoparticles. We show discrimination of metastatic LNs from benign LNs with two distinct features of NIRF imaging: increased tissue intensity and spatial distribution within the nodes. While previous studies have shown systemic detection of LN metastases with ICG-conjugated nanoparticles 40, this is the first study to characterize a microscopic pattern of nanomaterial accumulation within both benign and metastatic LNs. This nanoparticle-based LN imaging strategy has the potential to assist the diagnosis of LN status and offer clinical utility in the intraoperative visualization of at-risk LNs.

## Materials and Methods

### Polymeric micelles

UPS copolymers were synthesized following previously published procedures 23, 27. More specifically, ethylpropylaminoethyl methacrylate (EPA), dipropylaminoethyl methacrylate (DPA), and dibutylaminoethyl methacrylate (DBA) were used to synthesize UPS_6.9_, UPS_6.1_ and UPS_5.3_ copolymers by atom transfer radical polymerization (ATRP) from a polyethylene glycol (PEG)-bromide macroinitiator, respectively. ICG-sulfo-OSu (AAT Bioquest) was conjugated to primary amines at a molar ratio of three fluorophores per polymer in methanol for 24 h. Purification with discontinuous diafiltration in methanol using a 10 kDa regenerated cellulose ultrafiltration disc (Amicon Bioseparations) removes unconjugated ICG. ICG-conjugation is quantified by UV-Vis spectroscopy with the Shimadzu UV-1800 at polymer concentration of 10 μg/mL in methanol.

Purified ICG-copolymers in methanol are dispersed in deionized water ten-fold under sonication for micelle self-assembly. Micelles are purified in a 100 kDa centrifugal filter unit (Amicon Bioseparations) with three washes of deionized water. A stock concentration of micelles is maintained at 5.0 mg/mL. Micelle nanoparticles were characterized by dynamic light scattering (DLS) using the Malvern Zetasizer Nano ZS. Micelles were diluted to 0.1 mg/mL in phosphate buffered saline (PBS) at discrete pH (± 0.5 pH unit from the polymer pK_a_, Figure 1D). Additionally, ICG-fluorescence intensity was measured as a function of pH. Samples were imaged with the LICOR Pearl in the 800 nm channel at 85 μm resolution. A hand drawn region of interest (ROI) is placed over the center of a sample and copied to each sample to ensure consistent areas. An XY-plot shows the fluorescence response to pH. A non-linear regression in GraphPad Prism was used to fit the response to a best fit.

### Animal studies

The Institutional Animal Care and Use Committee at UT Southwestern (protocol number 2017-102139) approved all animal procedures and care. We utilized an orthotopic 4T1.2 BALB/cj model in eight week old mice 39. Implantation of 1 x 10^6^ cells in the fourth, right mammary fat pad resulted in consistent, spontaneous LN metastasis to ipsilateral axillary LNs as well as occasional metastasis to ipsilateral or contralateral cervical and inguinal LNs after 4-5 weeks of primary tumor growth. UPS nanoparticles were administered to 4T1.2-bearing BALB/cj mice intravenously in 0.9% saline at 1.0 mg/kg. All circulation times were 24 h between injection and euthanasia unless otherwise noted (Figure S9).

### Fluorescence imaging

Real-time fluorescence imaging is performed using an NIRF camera from OncoNano Medicine. Near-infrared excitation light was obtained from the Hamamatsu PDE. Emission light was filtered with a 860 ± 12 nm band-pass filter (ThorLabs) and focused with a 25 mm/F1.8 fixed focal length lens (Edmund Optics). Filtered emission wavelengths are detected with the Blackfly S USB3 camera (FLIR). Images were recorded at 4 fps unless otherwise specified. Individual LNs were resected under the guidance of fluorescence imaging system as well as a stereotactic microscope.

Quantitative NIRF imaging was performed with the LICOR Pearl Small Animal Imaging System. Image acquisition occurs at 85 μm resolution in the 800 nm channel. Quantification occurs in the Image Studio software, drawing ROI with the freehand tool. The median pixel intensity as well as LICOR signal was exported for each ROI. Moreover, fluorescent slides were scanned with the LICOR Odyssey imager at 21 μm resolution. Images are linked with the same filter for ease of comparison.

### Histology

After dissection, LN tissues were formalin-fixed, paraffin-embedded and sectioned in three 5.0 μm slices every 500 μm until tissue exhaustion. This led to three to four groups of three adjacent slides. The first slide is stained with hematoxylin and eosin using an automatic staining instrument (Dakewe). The second slide was used for NIRF imaging. The third adjacent slide was used for pan-cytokeratin immunohistochemistry. Heat-induced antigen retrieval was accomplished in Tris pH 9 for 17 min at 110 psi. Slides were blocked for 1 h with Mouse serum (Mouse on mouse blocking reagent, Vector Laboratories, Lot no. ZF0513). Anti-mouse pan-cytokeratin antibody (diluted 1:10; AE1/AE3 clone; ThermoFisher, Lot #UH2828424) in 2.5% normal horse serum (Vector Laboratories) incubation occurred for 30 min at room temperature. Detection of primary antibody was done for 10 min at room temperature with the Immpress Horse Anti-Mouse IgG Polymer Reagent (Mouse on mouse blocking reagent, Vector Laboratories, Lot no. ZF0418). The DAB substrate was added until color developed. Benign LNs are classified as pan-cytokeratin negative. Micro-metastases are defined as pan-cytokeratin positive clusters less than 2 mm in size. Macro-metastatic LNs are those with pan-cytokeratin positive clusters greater than 2 mm in size. A practicing pathologist (J.A.B.) interprets each pathology.

Immunohistochemistry staining enables visualization of spatial co-localization between nanoparticles and LN macrophages. BALB/cj mice (8 weeks old) were intravenously injected with 1.0 mg/kg nanoparticle solution in 0.9% saline. LNs were resected under guidance of the OncoNano NIRF camera system. LNs were embedded in OTC medium and frozen with liquid nitrogen. Frozen sections were sectioned at 12 μm at intervals of 500 μm. Sections were fixed in -20° C acetone for 10 min followed by 10 min of drying at room temperature. Next, sections were washed twice in 1x PBS for 5 min each. Blocking occurred with normal goat serum for 1 h. Aspiration of the blocking serum was followed by incubation of primary antibodies: FITC anti-mouse CD169 (1:125; Clone 3D6.112; Lot no. B271952), PE anti-mouse F4/80 (1:50; Clone BM8; Lot no. B199614), and APC anti-mouse CD11b (1:50; Clone M1/70; Lot no. B279418). All antibodies were multiplexed in PBS with 0.5% Tween and added to each tissue section. Incubation occurs overnight at 4 °C. Sections were washed three times in PBS for 5 min each. Mounting cover slips were used with Diamond Mount with DAPI (Invitrogen). Slides were imaged with the Keyence Automated Microscope.

### Statistical Analysis

LICOR signal and median CR values were grouped according to histological status. Each group (benign, micro-metastatic, and macro-metastatic) was analyzed with a one-way ANOVA for statistical difference of means. A Tukey multiple comparison assessed differences between the mean of each group. An 'ROC Curve' module with the 'Wilson/Brown' method was used in GraphPad Prism to compare discrimination between variables and groups. Sensitivity and specificity of the test is recorded using the Youden's J statistic. This statistic was maximized to determine the threshold for sensitivity and specificity.

## Supplementary Material

Supplementary figures and tables.

## Figures and Tables

**Table 1:** Receiver operating characteristic analysis of benign versus macro-metastatic LNs for UPS nanoparticles

Micelle	Groups	Variable	Sensitivity (%)	Specificity (%)	AUC
UPS_5.3_	Benign (n=17) Macro-met (n=13)	Signal	92.3	88.2	0.96
		Median CR	61.5	94.1	0.84
UPS_6.1_	Benign (n=20) Macro-met (n=15)	Signal	53.3	85.0	0.73
		Median CR	73.3	80.0	0.79
UPS_6.9_	Benign (n=12) Macro-met (n=14)	Signal	100	75	0.88
		Median CR	78.6	100	0.92

UPS: ultra-pH-sensitive; CR: contrast ratio; AUC: Area under the curve

## Abbreviations

LN: lymph node
SLNB: sentinel lymph node biopsy
UPS: ultra-pH-sensitive
NIRF: near-infrared fluorescence
ICG: indocyanine green
CR: contrast ratio
ROC: receiver operating characteristic
AUC: area under the curve
ROI: region of interest
