# Supplementary material for: Detection of Lymph Node Metastases by Ultra-pH-Sensitive Polymeric Nanoparticles
Source: Theranostics. 2020 Feb 10;10(7):3340–50. doi: 10.7150/thno.41239 (PMC7053196; doi:10.7150/thno.41239)
Supplement: Supplementary file 1 — Supplementary figures and tables. [file thnov10p3340s1.pdf]

Supplementary Information

Detection of Lymph Node Metastases by Ultra-pH-Sensitive Polymeric Nanoparticles

Zachary T. Bennett[1], Qiang Feng[1], Justin A. Bishop[2], Gang Huang[1], Baran D. Sumer[3*], and Jinming Gao[1,3*]

1. Harold C. Simmons Comprehensive Cancer Center, University of Texas Southwestern Medical Center, Dallas, TX, USA.

2. Department of Pathology, University of Texas Southwestern Medical Center, Dallas, Texas, USA

3. Department of Otolaryngology, University of Texas Southwestern Medical Center, Dallas, Texas, USA

*Corresponding authors: baran.sumer@utsouthwestern.edu; jinming.gao@utsouthwestern.edu

This file includes:

Table S1-S4

Figure S1-S10

Table S1. Characterization of PEG-*b*-(PR-*r*-AMA) diblock copolymers

Copolymer	M_n (kDa)[a]	Repeating Unit (x)[b]
PEG-*b*-P(DBA_x-*r*-AMA_y)	22.4	72
PEG-*b*-P(DPA_x-*r*-AMA_y)	22.0	66
PEG-*b*-P(EPA_x-*r*-AMA_y)	25.6	118

[a]Number-averaged molecular weights were determined by GPC using THF as the eluent. [b]Repeating unit was calculated based on integrations of -CH_2-O- groups on side chains to the methylene groups on PEG using ^{1}H NMR.

Table S2. Measurement of conjugation efficiency and quantum yields of dye-conjugated copolymers

PR-Dye	Dye Conjugation		
	Dye per polymer (x)[a]	Efficiency (%)[a]	ON/OFF Ratio[b]
P(DBA_{70}-*r*-ICG_x)	1.9	0.63	56
P(DPA_{65}-*r*-ICG_x)	2.0	0.62	59
P(EPA_{115}-*r*-ICG_x)	1.8	0.76	39

[a]Determined by a standard curve base on UV-Vis spectroscopy of the free ICG in methanol. [b]Determined by the ICG fluorescence emission in 1x PBS using the LICOR Pearl Imager.

Table S3. Characterization of PEG-b-(PR-r-Dye) nanoprobes.

PR-Dye	Particle Size (nm)[a]	pH_t[b]	$\Delta pH_{10\text{-}90\%}$[c]
$UPS_{5.3}$-ICG	28.5 ± 1.5	5.3	0.28
$UPS_{6.1}$-ICG	24.0 ± 2.1	6.1	0.33
$UPS_{6.9}$-ICG	23.4 ± 2.5	6.9	0.24

[a]Number-based size determined by dynamic light scattering. [b]Determined by ICG fluorescence using the LICOR Pearl Imager. [c]Determined by NaOH-titration.

Table S4. Receiver operating characteristic analysis of benign versus micro-metastatic LNs for UPS nanoparticles

Micelle	Groups	Variable	Sensitivity (%)	Specificity (%)	AUC
$UPS_{5.3}$	Benign (n=17)<br>Micro-met (n=39)	Signal	69.2	58.8	0.67
		Median CR	87.2	58.8	0.64
$UPS_{6.1}$	Benign (n=20)<br>Micro-met (n=10)	Signal	50.0	75.0	0.58
		Median CR	90.0	70.0	0.76
$UPS_{6.9}$	Benign (n=12)<br>Micro-met (n=17)	Signal	64.7	66.7	0.60
		Median CR	55.6	66.7	0.55

UPS = ultra-pH-sensitive; CR: contrast ratio; AUC = area under the curve

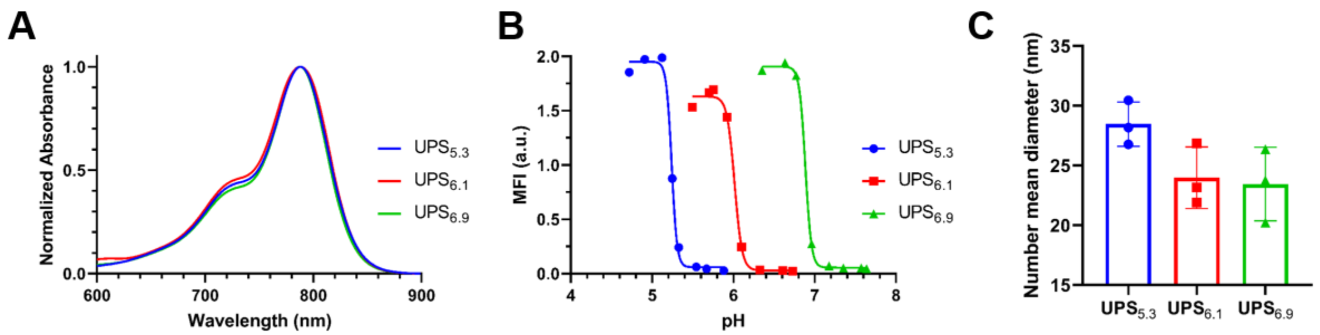

Figure S1. In vitro characterization of UPS-ICG nanoparticles. (A) UPS-ICG nanoparticles absorb near-infrared light at λ_{max} of 788 nm. (B) Raw mean fluorescence intensity of UPS-ICG nanoparticles measured by LICOR Pearl 800 nm channel. (C) The number mean diameter of UPS-ICG nanoparticles measured by dynamic light scattering.

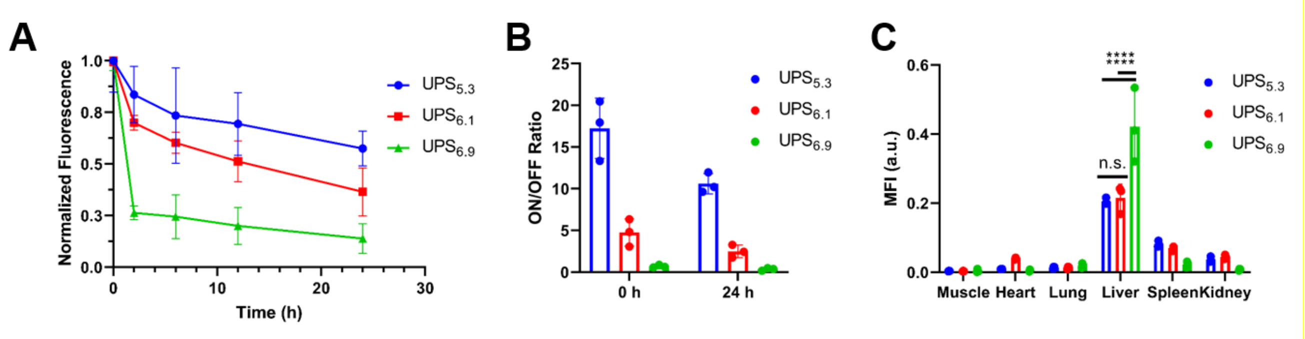

Figure S2. Pharmacokinetics and organ distribution of UPS nanoparticles in Balb/cj mice. (A) Pharmacokinetics of UPS-ICG fluorescence in collected plasma. Plasma is acidified to show the 'ON' state of the nanoparticles. Plasma fluorescence is normalized to fluorescence at time 0 h, controlling for differences between UPS compositions. (B) Acidified plasma fluorescence is normalized to the collected plasma, showing the 'ON/OFF Ratio'. (C) Ex vivo imaging of organs after 24 h circulation of UPS nanoparticles.

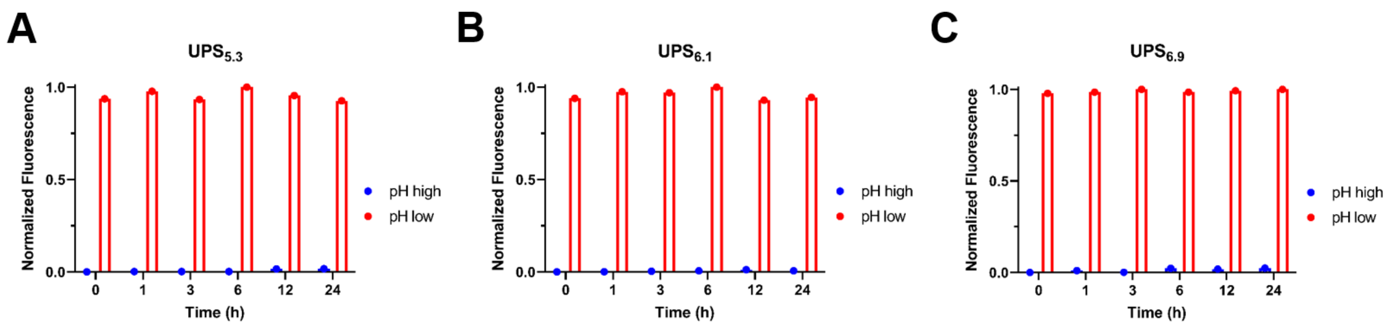

Figure S3. Serum stability of the UPS nanoparticle library. (A) $UPS_{5.3}$ (B) $UPS_{6.1}$ (C) $UPS_{6.9}$ all show quenched fluorescence in serum as measured by the LICOR Pearl imager. After decreasing pH with addition of acid, the sample fluorescence increased many fold.

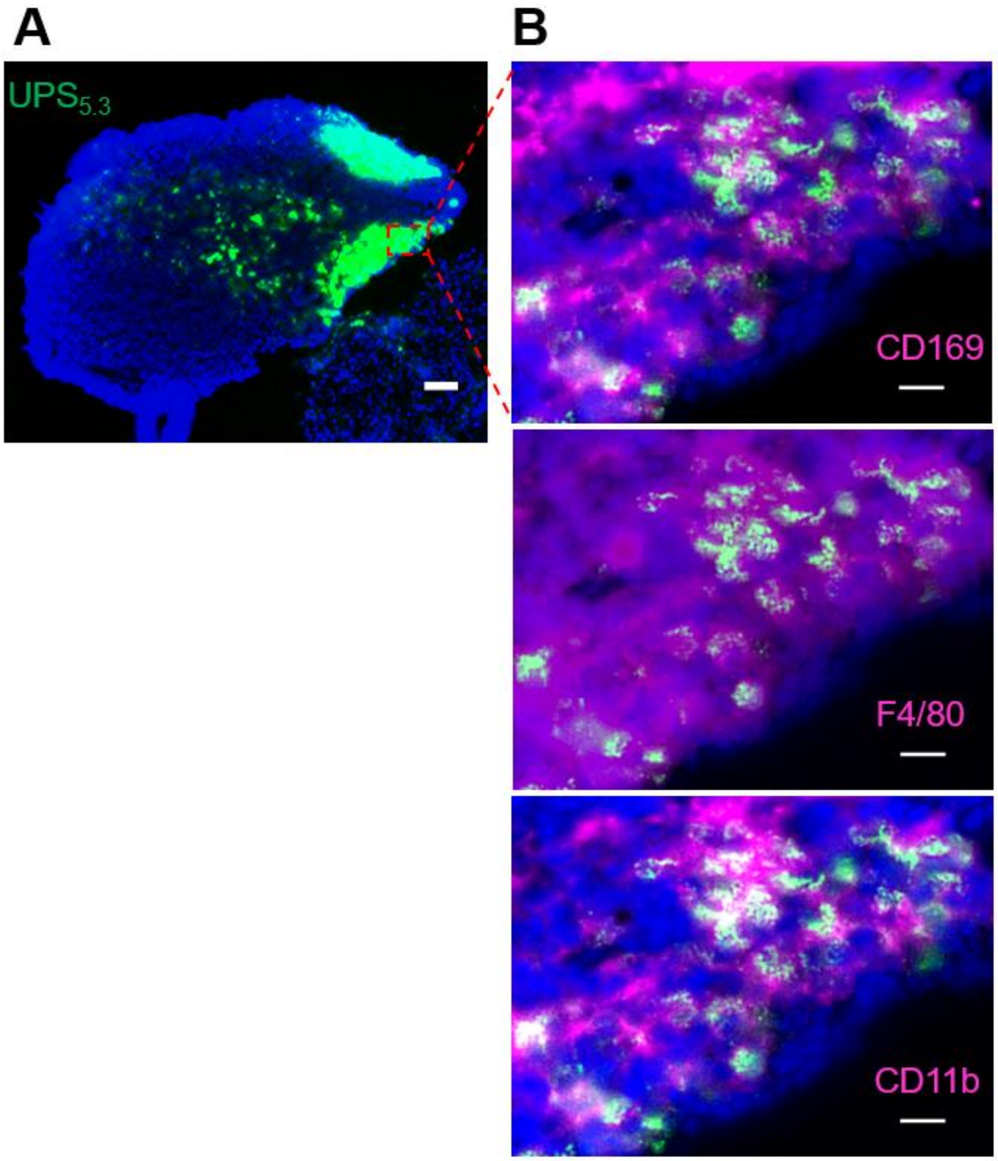

Figure S4. Lymph node-resident macrophages show co-localization with ICG signal within the lymph node. (A) Regions in LNs from $UPS_{5.3}$ administered mice show $UPS_{5.3}$-ICG fluorescence in the LN sinusoids. Scale bar is 50 µm. (B) ICG fluorescence co-localizes with all three macrophage markers CD169, F4/80, and CD11b. The $CD169^{+}$/$F480^{+}$/$CD11b^{+}$ macrophage is characterized as medullary sinus macrophage. Scale bar is 10 µm.

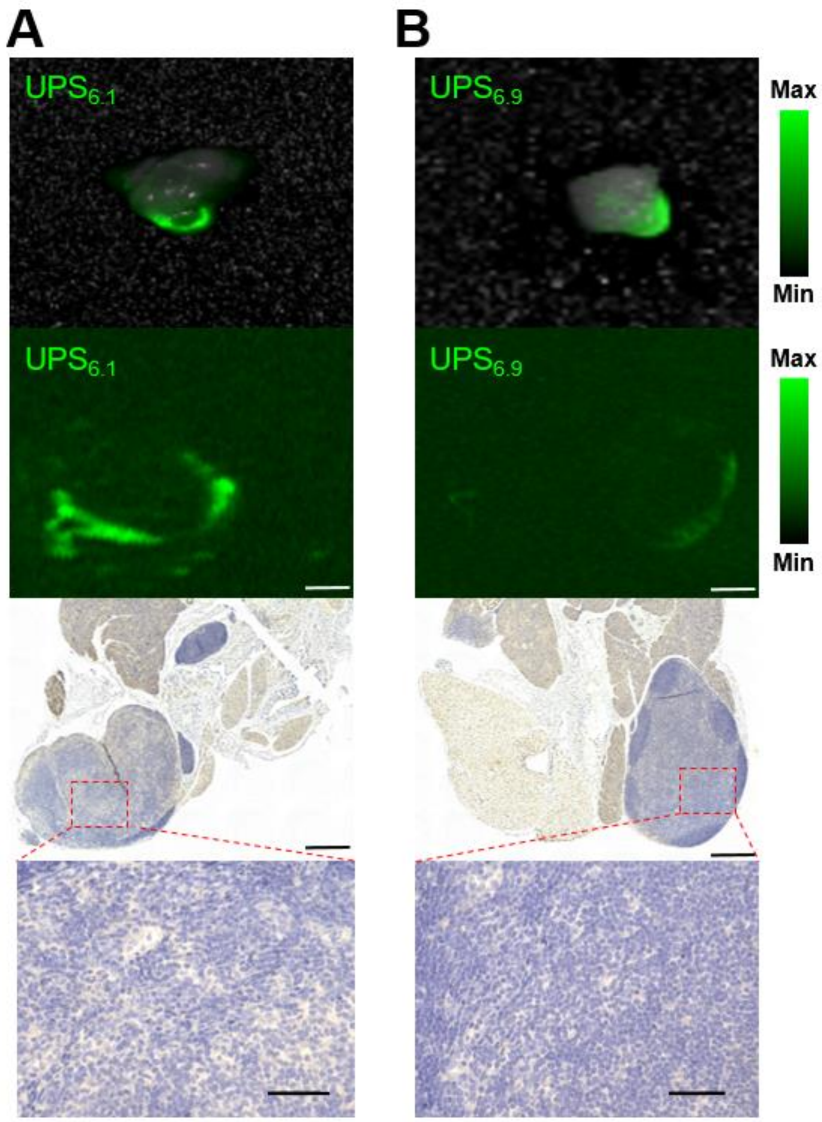

Figure S5. Benign LNs from UPS$_{6.1}$ and UPS$_{6.9}$ administered mice show edge-localization. (A) NIRF imaging of gross LN tissue shows distinct pattern of UPS$_{6.1}$-ICG fluorescence around the edge of the benign LN. This spatial localization is confirmed with microscopic imaging of formalin-fixed, paraffin-embedded tissue sections. The adjacent tissue section is used for staining of pan-cytokeratin. No distinct cytokeratin puncta are found in the benign LN. (B) UPS$_{6.9}$-ICG shows similar nanoparticle distribution around LN edges, but the intensity is lower due to less available biodistribution. Upper scale bar is 300 μm. Lower scale bar is 50 μm.

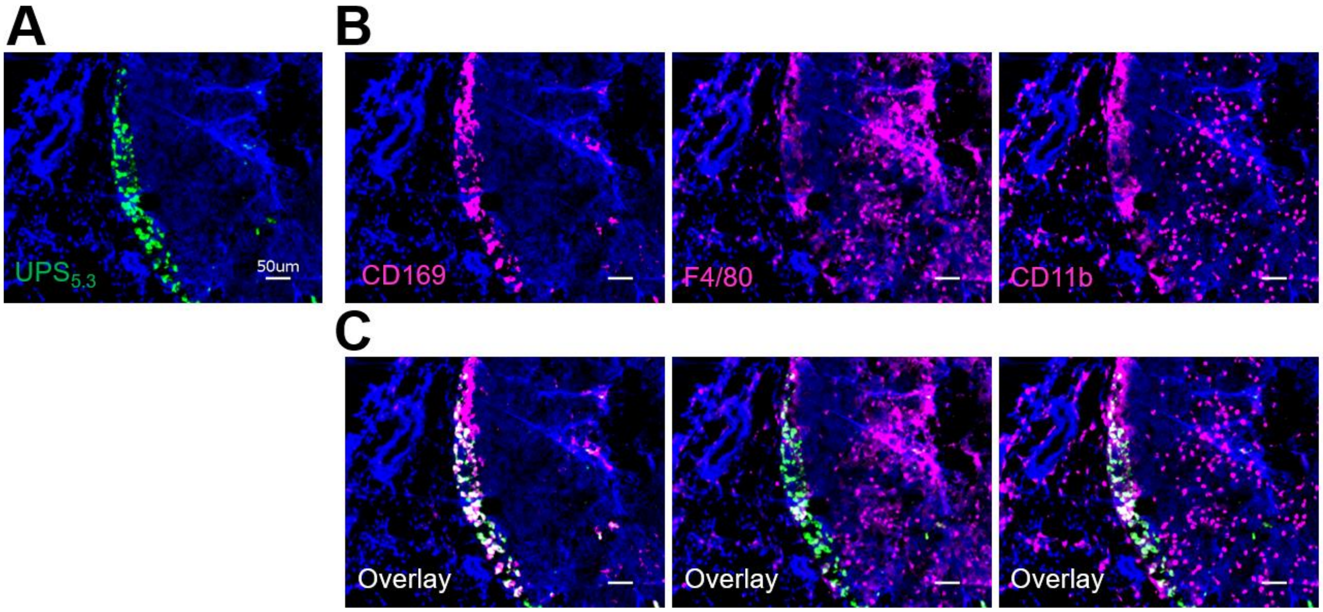

Figure S6. Lymph node-resident macrophage from tumor-bearing mice internalize $UPS_{5.3}$ nanoparticles. (A) ICG fluorescence in benign LNs from 4T1.2 tumor-bearing mice appears in distinct clusters in lymphoid tissue. (B) $CD169^+$ macrophages cluster at an edge within the lymphoid tissue. $F4/80^+$ macrophages appear highly localized in the LN cortex. This suggests a more migratory phenotype compared to tumor naïve mice. $CD11b^+$ macrophages are present both within the LN as well as in the surrounding tissue. This is markedly different from tumor naïve mice, which only show $CD11b^+$ cells within the LN. Functionally, CD11b mediates inflammatory responses by directing leukocyte migration. This supports the notion of migratory leukocyte populations in the lymphoid tissue. (C) ICG fluorescence in the benign LNs mostly co-localizes with $CD169^+$ LN resident macrophages located in the LN sinusoids.

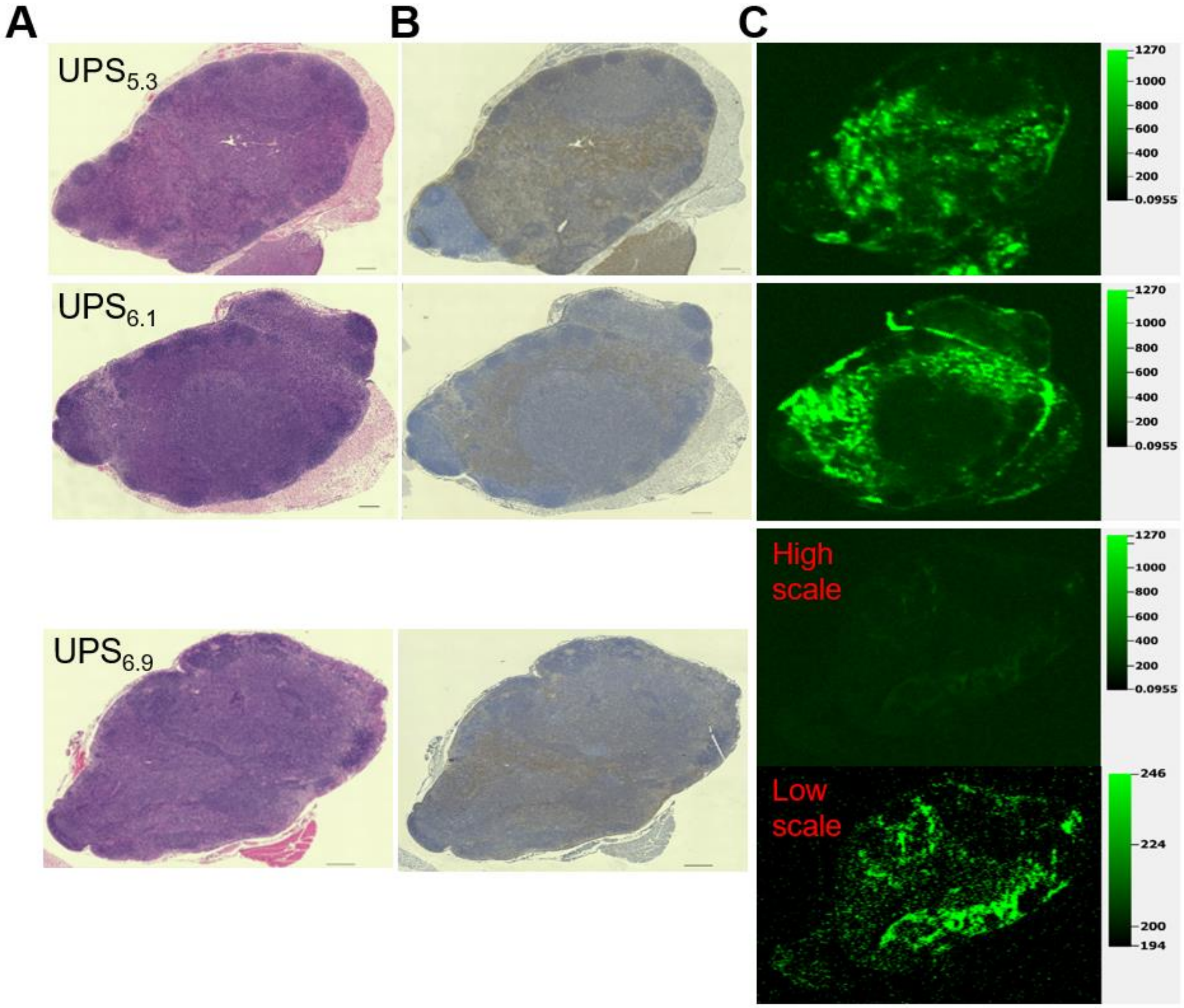

Figure S7. UPS nanoparticle accumulation in macro-metastatic lymph nodes. (A) H&E staining of axillary lymph node shows enlarged nodes. (B) Anti-cytokeratin immunohistochemistry staining reveals presence of cancer foci in the LNs. (C) Near infrared fluorescence scanning of tissue sections reveals $UPS_{5.3}$-ICG and $UPS_{6.1}$-ICG accumulate in areas with pan-cytokeratin expression. $UPS_{6.9}$-ICG displays a much lower fluorescence intensity at the same fluorescent scale as $UPS_{5.3}$ and $UPS_{6.1}$. Low scale display show $UPS_{6.9}$ accumulation in pan-cytokeratin positive regions. Scale bar is 300 μm.

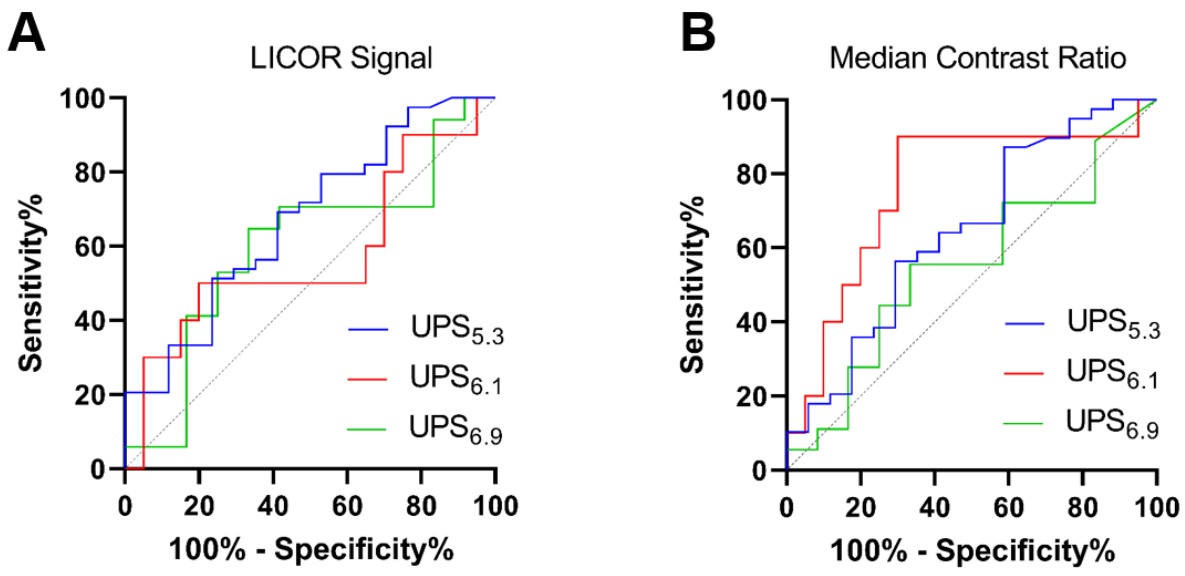

Figure S8. Receiver operating characteristic (ROC) analysis of micro-metastatic lymph node detection by UPS nanoparticles. (A) ROC curve comparing benign and micro-metastatic nodes using LICOR signal variable. No micelle group displays significant discrimination of benign from micro-metastatic LNs. (B) ROC curve comparing benign and micro-metastatic nodes using median CR variable. Compared to metastatic LNs (Figure 7), UPS nanoprobes have less discriminating power to resolve micro-metastatic nodes over benign nodes.

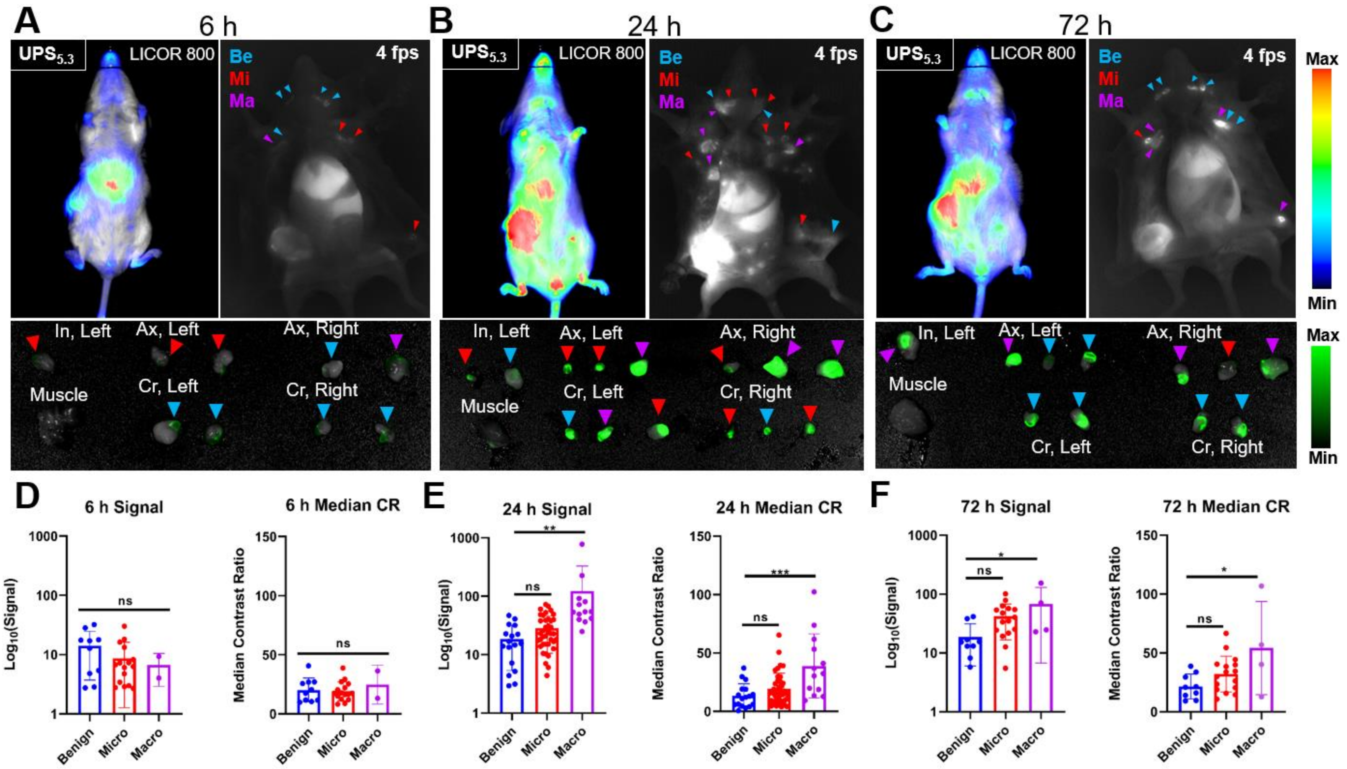

Figure S9. Varying circulation time after UPS$_{5.3}$ injection in lymph node metastasis detection. (A) A representative 4T1.2-bearing BALB/cj mouse 6 h after injection of UPS$_{5.3}$-ICG shows NIRF detection of the primary tumor with whole body imaging. Benign (Be), micro-metastatic (Mi), and macro-metastatic (Ma) LNs, do not show high fluorescence intensity. Inguinal (In), axillary (Ax), and cervical (Cr) LNs are resected with some fluorescence guidance but also under white light. (B) NIRF imaging of a mouse 24 h after injection of UPS$_{5.3}$-ICG administered mice shows delineation of the primary tumor and LNs, with bright metastatic LNs. (C) Mice 72 h after injection of UPS$_{5.3}$-ICG shows comparable accumulation in macro-metastatic LNs, but there is less whole body background. (D) 6 h UPS$_{5.3}$ signal and median CR of classified tissue shows no significance between metastatic and benign LNs. The signal variable is much lower in intensity compared to 24 h and 72 h. Statistical analysis is done with one-way ANOVA followed by Tukey's multiple comparisons test (*$P < 0.033$, **$P < 0.0021$, ***$P < 0.0002$). (E) 24 h UPS$_{5.3}$ signal and median CR of classified tissue shows significance between metastatic and benign LNs. (F) 72 h UPS$_{5.3}$ signal and median CR of classified tissue shows significance between macro-metastatic and benign LNs.

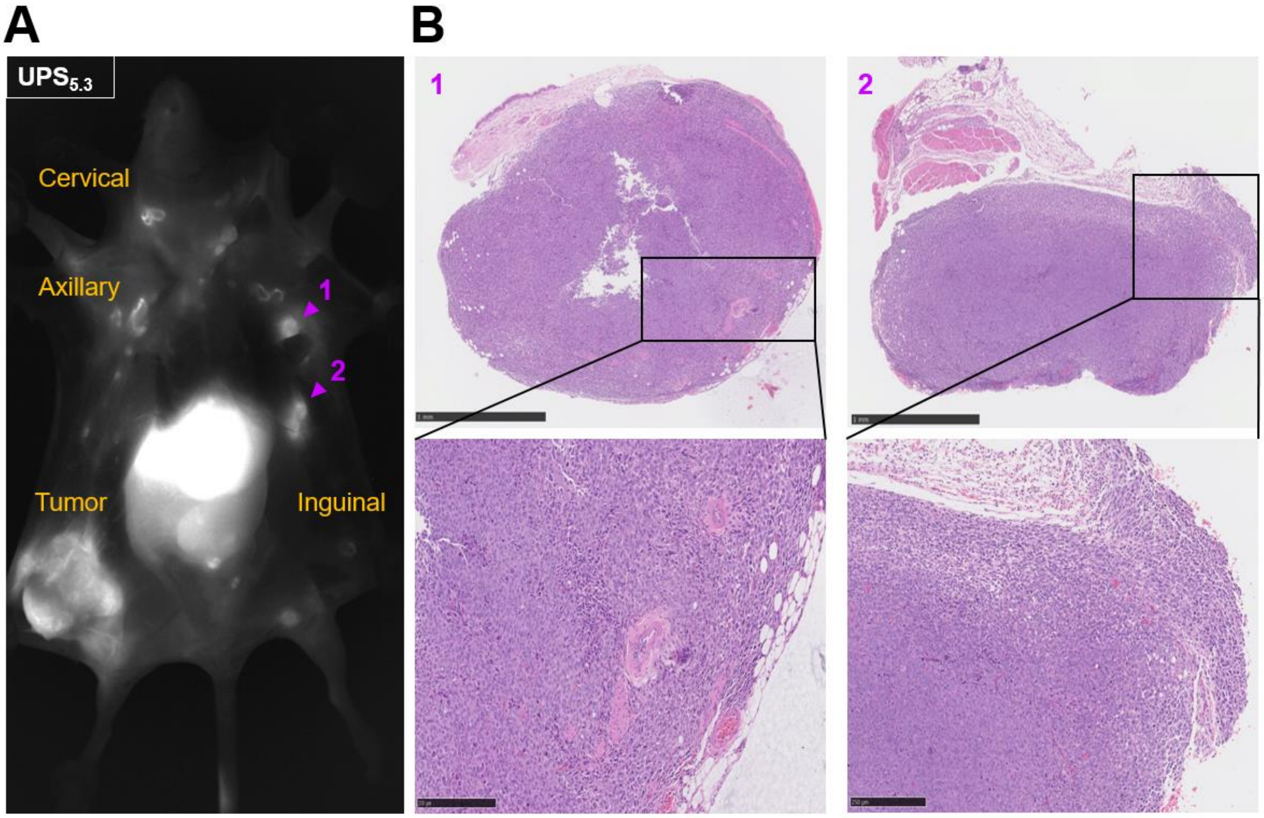

Figure S10. Regional metastases in-transit are detectable with real-time NIR fluorescence imaging. (A) NIRF imaging with $UPS_{5.3}$-ICG identifies tumor foci (1 and 2) outside of the regional LN basin. These tumor foci appear bright with homogenous fluorescence signals across the lesion. Each tumor is resected under fluorescence guidance. (B) The resected tissue is FFPE prepared and sectioned in thin slices. H&E staining shows clear cancer foci with access to vasculature. Above scale bar is 1.0 mm. Lower scale bar is 200 µm.
